# Neutrophil Extracellular Traps in SARS-CoV2 Related Pneumonia in ICU Patients: The NETCOV2 Study

**DOI:** 10.3389/fmed.2021.615984

**Published:** 2021-02-23

**Authors:** Mathieu Godement, Jaja Zhu, Charles Cerf, Antoine Vieillard-Baron, Agathe Maillon, Benjamin Zuber, Valérie Bardet, Guillaume Geri

**Affiliations:** ^1^Medical Intensive Care Unit, Ambroise Paré Hospital, APHP, Paris, France; ^2^Paris Saclay University, Saint-Aubin, France; ^3^Biological Hematology Department, Ambroise Paré Hospital, APHP, Paris, France; ^4^Medico-Surgical Intensive Care Unit, Foch Hospital, Paris, France; ^5^INSERM UMR 1018, Clinical Epidemiology Team, CESP, Paris, France; ^6^FHU SEPSIS (Saclay Endeavour to PersonnaliSe Interventions for Sepsis), Paris Saclay University, Saint-Aubin, France

**Keywords:** COVID19, SARS-CoV2, neutrophils extracellular traps neutrophils extracellular traps, coronavirus, pneumonia

## Abstract

**Background:** Severe acute respiratory syndrome coronavirus 2 (SARS-CoV-2) is a poorly understood disease involving a high inflammatory status. Neutrophil extracellular traps (NETs) have been described as a new pathway to contain infectious diseases but can also participate in the imbalance of the inflammatory and the coagulation systems. NETs could be a therapeutic target in COVID-19 patients.

**Methods:** Consecutive patients with SARS-CoV2 related pneumonia admitted to the intensive care unit were included in a prospective bicentric study. Neutrophil extracellular trap concentrations were quantified in whole blood samples at day-1 and day-3 by flow cytometry. The primary outcome was the association between the blood NET quantification at ICU admission and the number of days with refractory hypoxemia defined by a PaO_2_/FIO_2_ ratio ≤100 mmHg.

**Results:** Among 181 patients admitted to the ICUs for acute respiratory failure related to SARS-CoV2 pneumonia, 58 were included in the analysis. Patients were 62 [54, 69] years old in median, mostly male (75.9%). The median number of days with severe hypoxemia was 4 [2, 6] days and day-28 mortality was 27.6% (*n* = 16). The blood level of NETs significantly decreased between day-1 and day-3 in patients who survived (59.5 [30.5, 116.6] to 47 [33.2, 62.4] *p* = 0.006; 8.6 [3.4, 18.0] to 4 [1.4, 10.7] *p* = 0.001 and 7.4 [4.0, 16.7] to 2.6 [1.0, 8.3] *p* = 0.001 for MPO+, Cit-H3+, and MPO+ Cit-H3+ NETs, respectively) while it remained stable in patients who died (38.4 [26.0, 54.8] to 44.5 [36.4, 77.7] *p* = 0.542; 4.9 [1.3, 13.0] to 5.5 [2.8, 6.9] *p* = 0.839 and 4 [1.3, 13.6] to 2.7 [1.4, 4.5] *p* = 0.421 for MPO+, Cit-H3+, and MPO+ Cit-H3+ NETs, respectively). In multivariable negative binomial regression, the blood level of MPO+ NETs was negatively associated with the number of days with severe hypoxemia within 7 days (0.84 [0.73, 0.97]), while neither Cit-H3+ NETs nor double-positive NETs were significantly associated with the primary outcome.

**Conclusion:** The whole blood level of NETs at day-1 was negatively associated with the number of days with severe hypoxemia in patients admitted to the intensive care unit for SARS-CoV2 related pneumonia. The lack of decrease of the blood level of NETs between day-1 and day-3 discriminated patients who died within day-28.

## Introduction

Neutrophils extracellular traps (NETs) are the result of neutrophil extrusion of extracellular fibers composed of DNA, histones, and granule-derived proteins released by neutrophils, which trap and kill extracellular pathogens (1). NETosis is triggered by several metabolic pathways including NADPH oxidase (2) and peptidylarginine deaminase 4 (PAD4)-induced citrullination of histones (3) which converge to mediate the cellular process of chromatin decondensation necessary for NET release from neutrophils. The relative importance of NADPH oxidase and PAD4 for completion of NETosis may be dictated by the cellular stimulus. NETs are important players in the genesis, growth and resolution of the coagulation cascade (4, 5) and may participate in the imbalance of inflammation and coagulation in sepsis (6). This is particularly well-described in the context of acute respiratory distress syndrome (ARDS) related to bacterial (7–10) or influenza pneumonia (11–13), linked to the massive invasion of alveoli by an inflammatory infiltrate containing neutrophils, monocytes, macrophages, altered epithelial cells and numerous pro-inflammatory markers (14) Blood and alveoli levels of NETs in ARDS patients strongly correlate with the severity of respiratory disease (7, 15).

Respiratory failure is the leading cause of death in the coronavirus disease 19 (COVID-19) involving simultaneously and at different degrees lung injury related to viral invasion, pulmonary thrombosis and cytokine storm (16–18). Neutrophils may play a cornerstone role in the pathogenesis of the most severe cases (19). Increased counts of blood neutrophils and a high neutrophil-to-lymphocyte ratio are associated with severe respiratory disease and worse outcomes in this setting (20, 21). Interestingly, lung tissue microscopic examination evidenced neutrophilic infiltration in pulmonary capillaries, extravasation of neutrophils into the alveolar space, and neutrophilic mucositis as well as alveolar capillary microthrombi (22, 23).

Taken together, NETs may represent an interesting factor that could be associated with both viral pneumonia and thrombosis. Thus, we decided to investigate the association between the whole blood levels of NETs at ICU admission and the respiratory failure evolution toward refractory hypoxemia in ICU patients with SARS-COV2 related pneumonia.

## Materials and Methods

### Patients and Data Collection

All consecutive patients admitted to the ICU meeting the following inclusion criteria were included: age ≥18 years, SARS-CoV2 related pneumonia documented at least on one of the following criteria: SARS-CoV2 positive PCR on a sample of the upper and/or lower airways and/or typical CT scan lung pattern, as previously described (24). Patients were not included in case of pregnancy, guardianship or curatorship or if they had signed an opposition form.

Demographics, clinical and laboratory variables were recorded during intensive care unit stay as well as the use of adjuvant therapies for ARDS, the need for hemodialysis or vasopressors, corticosteroid administration, the occurrence of thrombotic events (both venous thromboembolism and arterial thrombotic complications), the number of ventilator- and organ failure–free days at day 28, and the duration of mechanical ventilation. Vital status at day-28 was also recorded.

The electronic CRF (e-CRF) developed by Clinfile were used for data collection from each center.

### Controls

Healthy volunteer blood donors were used as “controls.”

### Outcomes

The primary clinical endpoint was the association between the blood level of NETs at ICU admission and the number of days with refractory hypoxemia defined by a PaO_2_/FIO_2_ ratio lower than 100.

Secondary outcomes included the association between the blood NET measurements at ICU admission and (1) the need for orotracheal intubation, (2) criteria for ARDS in mechanically ventilated patients according to the Berlin classification (25), (3) admission severity scores, i.e., the simplified acute physiology score (SAPS II) and the sequential organ failure assessment (SOFA) (26–28), (4) the blood levels of inflammatory biomarkers [procalcitonin and C-reactive protein (CRP)], (5) day-28 all-cause mortality, (6) the number of mechanical ventilation free days (number of days without mechanical ventilation, patients who died on mechanical ventilation being rated zero), (7) the main markers of blood hemostasis [i.e., prothrombin ratio, activated partial thromboplastin time (aPTT) ratio, blood platelets level, D-dimers, antithrombin-III, C protein as well as fibrinogen] and anticoagulation regimens, and (8) the occurrence of thrombotic events (both venous thromboembolism and arterial thrombotic complications) and pulmonary circulatory failure. Pulmonary circulatory failure was assessed by critical care echocardiography; right ventricular dysfunction was retained if there was a right ventricular dilation defined as a ratio of end diastolic area of right ventricle on left ventricle >0.6 or an *acute cor pulmonale* (29, 30).

### NET Quantification

NETs were quantified in whole blood samples at day-1 and day-3 and bronchoalveolar fluid (BAL) (in intubated patients performed at the time of intubation) by flow cytometry according to an in-house technique adapted from Gavillet and Masuda et al. (31, 32). As nothing was known regarding SARS-COV2-induced NETosis, we quantified several NET sub populations: single positive MPO+, single positive Cit-H3+ and double positive MPO+ Cit-H3+. Staining was performed with a “lysis-no wash” protocol to preserve NET integrity. 50 μL from whole blood sample or 1 mL from BAL were stained with the DNA-dye Hoechst 34580 (Life Technologies, Courtaboeuf, France) and Histone H3 (citrulline R2 + R8 + R17) rabbit polyclonal antibody (Abcam, Amsterdam, Netherlands) according to manufacturers' instructions and incubated 30 min at 37°C. Cells were then stained with SYTOX Green Dead Cell stain (Life Technologies, Courtaboeuf, France), Goat Anti-Rabbit IgG H&L-APC (Abcam), MPO-PE (Becton Dickinson, San Jose, CA, USA) according to manufacturers' instructions and incubated 30 min at room temperature. Red blood cell lysing was then performed using BD Pharm Lyse (Becton Dickinson) according to manufacturer's instructions. Data were acquired using a Lyric cytometer (Becton Dickinson) and analyzed using the Kaluza software (Beckman Coulter, Roissy, France). Positivity thresholds for MPO and Cit-H3 were assessed using negative isotypic controls.

**Figure 1:**
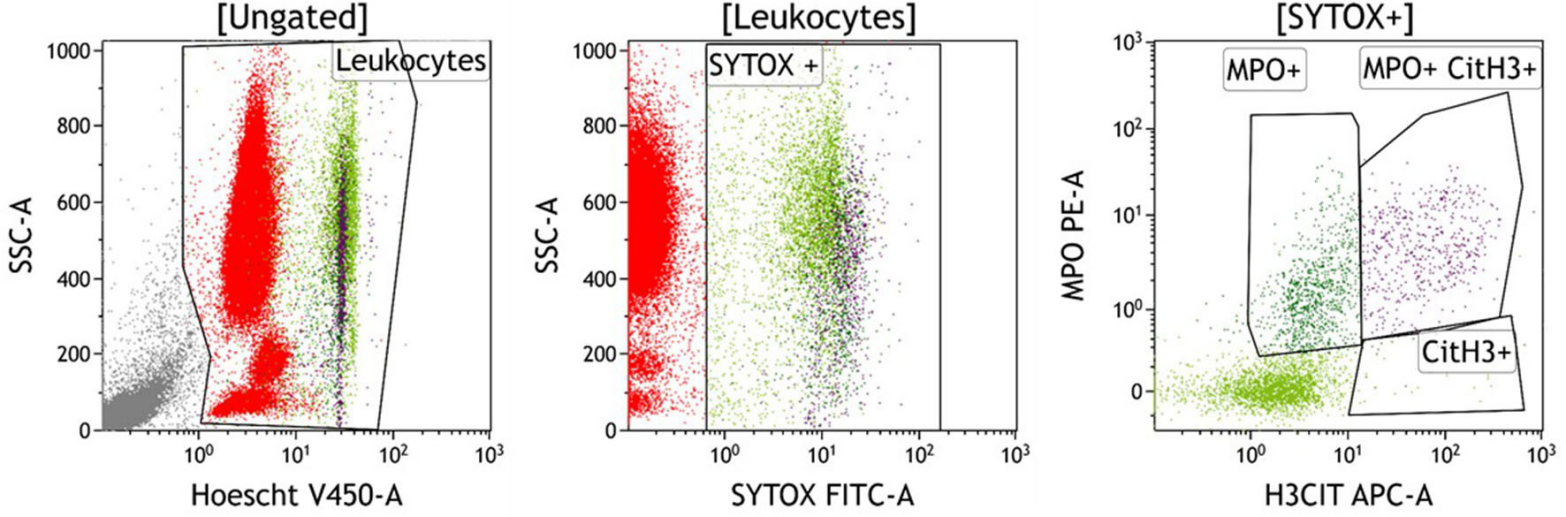
Typical staining of NETs in blood sample of a patient with SARS-COV2. After gating of nucleated cells (Leukocytes) with the DNA marker Hoechst 34580 (Plot A), SYTOX positive cells (ie cells with compromised plasma membranes) were isolated within the Leukocytes gate (Plot B). Single-positive (MPO+ or Cit-H3+) and double-positive (MPO+ Cit-H3+) NETs were then quantified in the SYTOX+ population (Plot C), respectively at 0.90, 0.03, and 0.52% for this patient.

The analytical strategy of NETs quantification is depicted in Figure 1. Nucleated cells were isolated using a Hoechst 34580 labeling and SYTOX Green positive cells (SYTOX+ cells) were gated as previously described (32), Single-positive (MPO+ or Cit-H3+) and double-positive (MPO+ Cit-H3+). NETs were then quantified as a percentage of nucleated cells and absolute values were calculated using leukocyte count or BAL cell count.

### Statistical Analysis

Data were described according to the primary outcome by the *n* (percentage) for the qualitative and median [interquartile] variables for the quantitative variables. Qualitative variables were compared using a Pearson chi-2 test or an exact Fisher test as appropriate. Quantitative variables were compared using a non-parametric Mann-Whitney.

The data were also described according to the blood NETs quantification on day-1 (divided into tertiles). The comparison between groups were performed using the Jonckheere-Terpstra test to take into account the order of categories (33, 34).

Pearson's correlation coefficients were calculated to assess the correlation between the day-1 blood level of NETs and hemostasis markers as listed above.

A negative binomial regression model was used to obtain an estimate of the effect of the blood level of NETs at day-1 on the number of days with severe hypoxemia within day-7. The exponential form of the estimate is called the incidence rate ratio. We included in the multivariable model all the relevant variables as well as the blood level of NETs. Relevant interactions were tested.

A *p*-value < 0.05 was considered significant. The analysis was performed using R (35).

### Ethics

The present study was approved by the Research Ethics Board of the Foch Hospital (Suresnes, France, n° 20-04-01) on April 6th, 2020.

## Results

### Baseline Characteristics

Baseline characteristics of the 58 patients included in the analysis are shown in Table 1. Patients were 62 [iqr 54, 69] years old in median, mostly male (75.9%). COVID-19 diagnosis relied on a positive PCR in 55 (94.8%) cases and an abnormal lung CT-scan in all cases. ICU admission occurred after 9 [iqr 7, 11] days in median after symptom onset. Median Charlson score was 3 [1, 3]. Median SAPS2 score was 32 [iqr 26, 46] at ICU admission.

**Table 1:** Baseline characteristics of the 58 included patients according to the number of days with severe hypoxemia within day-7.

	**[0,3) days (*N* = 20)**	**[3,6) days (*N* = 18)**	**[6,8) days (*N* = 20)**	**All patients (*N* = 58)**	***P*-value**
Age, years	59.0 [45.0; 71.0]	63.0 [57.0; 69.0]	61.5 [56.0; 68.5]	62.0 [54.0; 69.0]	0.757
Male gender	12 (60.0)	15 (83.3)	17 (85.0)	44 (75.9)	0.122
Body mass index, kg/m^2^	25.5 [24.5; 30.5]	28.7 [24.7; 30.1]	27.5 [25.0; 37.2]	27.5 [24.6; 31.0]	0.496
Positive PCR SARS-CoV2	19 (95.0)	17 (94.4)	19 (95.0)	55 (94.8)	0.996
Lung-CT scan involvement					0.597
<25%	4 (20.0)	0 (0.0)	1 (5.6)	5 (9.1)	
25–50%	4 (20.0)	3 (17.6)	4 (22.2)	11 (20.0)	
50–75%	8 (40.0)	8 (47.1)	6 (33.3)	22 (40.0)	
>75%	4 (20.0)	6 (35.3)	7 (38.9)	17 (30.9)	
Time interval between symptoms onset and ICU admission, days	9.0 [6.5; 12.0]	10.0 [7.0; 11.0]	8.0 [7.0; 10.0]	9.0 [7.0; 11.0]	0.866
Pulmonary embolism	2 (11.8)	5 (27.8)	5 (26.3)	12 (22.2)	0.454
Deep-vein thrombosis	1 (5.6)	3 (17.6)	2 (11.1)	6 (11.3)	0.529
Anti-inflammatory drugs before admission	0 (0.0)	0 (0.0)	1 (5.0)	1 (1.7)	0.380
Corticosteroids before admission	2 (10.0)	1 (5.6)	4 (20.0)	7 (12.1)	0.371
Immunosuppressive drugs before admission	3 (15.0)	1 (5.6)	1 (5.0)	5 (8.6)	0.454
Charlson comorbidities score	2.5 [1.0; 4.0]	2.5 [2.0; 3.0]	3.0 [1.0; 3.0]	3.0 [1.0; 3.0]	0.943
SAPS2 score	34.0 [22.0; 50.5]	28.5 [25.0; 34.0]	35.5 [27.5; 51.0]	32.0 [26.0; 46.0]	0.426
SOFA score	3.5 [3.0; 5.5]	4.5 [4.0; 5.0]	5.0 [4.0; 5.0]	4.0 [3.0; 5.0]	0.245
Blood leucocytes, G/L	12.1 [8.1, 12.7]	10.2 [8.5, 12.4]	8.4 [4.5, 10.9]	9.8 [6.9, 12.6]	0.102
Blood polymorphonuclear cells, G/L	9.2 [5.7, 11.0]	8.7 [6.7, 11.5]	6.8 [3.2, 9.1]	8.2 [5.4, 10.8]	0.096
Blood lymphocytes, G/L	0.8 [0.7, 1.3]	0.7 [0.5, 1.0]	0.6 [0.4, 1.0]	0.7 [0.6, 1.1]	0.130
C-reactive protein, mg/L	183 [140, 305]	188 [125, 242]	183 [128, 250]	187 [127, 255]	0.731
Procalcitonin, mg/L	0.6 [0.2, 2.0]	0.4 [0.2, 1.7]	0.4 [0.2, 2.7]	0.4 [0.2, 2.0]	0.994
Fibrinogen, g/L	6.7 [6.5, 8.4]	6.3 [5.1, 6.6]	6.8 [5.4, 8.8]	6.7 [5.6, 8.4]	0.213

### NETosis

We observed a strong correlation between day-1 whole blood level of NETs and blood leucocytes (Supplementary Figures 1, 2) at day-1 and day-2. Blood levels of NETs were similar in controls and in SARS-CoV2 patients except for MPO+ CIt-H3+ NETs which were significantly higher in COVID-19 patients (Supplementary Figure 3).

### Primary Outcome

Overall we observed a median number of days with severe hypoxemia (PaO_2_/FIO_2_ <100 mHg) of 4 [iqr 2, 6] days. No difference was observed in the three tertiles of number of days with severe hypoxemia (Table 1). The blood level of NETs did not differ either across these three tertiles (Supplementary Table 1). Conversely, we did not observe any significant association between quartiles of blood levels of NETs at day-1 and the number of days with severe hypoxemia (Supplementary Table 2).

In multivariable negative binomial regression, after adjustment for age, gender, SAPS II and Charlson scores, lung CT-scan lesions and time interval between onset of symptoms and ICU admission, MPO+ NETs were negatively associated with the number of days with severe hypoxemia within 7 days, while neither Cit-H3+ NETs nor MPO+ Cit-H3+ NETs were significantly associated with the primary outcome (Table 2).

**Table 2:** Association between blood levels of NETs at day-1 and number of days with severe hypoxemia within day-7.

	**Incidence rate ratio [95% confidence interval]**		
Age, per year	1.02 [1.00, 1.03]	1.02 [1.00, 1.04]	1.02 [1.00, 1.04]
Male gender	1.77 [1.18, 2.64]	1.91 [1.25, 2.91]	1.93 [1.27, 2.96]
SAPSII, per unit increase	1.01 [0.99, 1.02]	1.01 [0.99, 1.02]	1.01 [0.99, 1.02]
Charlson comorbidities score	0.84 [0.73, 0.97]	0.85 [0.74, 0.99]	0.85 [0.73, 0.98]
Lung CT-scan lesions^*^	1.18 [1.00, 1.39]	1.22 [1.02, 1.46]	1.22 [1.03, 1.46]
Time interval between onset of symptoms and ICU admission, days	0.98 [0.93, 1.02]	0.98 [0.93, 1.02]	0.97 [0.93, 1.02]
**NETs**			
MPO+, per 50/μmol increase	0.84 [0.73, 0.97]	–	–
Cit-H3+, per 5/μmol increase	–	0.99 [0.71, 1.37]	–
MPO+ Cit-H3+ per 5/μmol increase	–	–	0.99 [0.94, 1.04]

*Multivariable negative binomial regression was performed adjusting for age, gender, SAPS II and Charlson score, lung CT-scan lesions and time interval between onset of symptoms and ICU admission. The interpretation of the incidence rate ratio is as follows: an increase of 50/μmol of the blood level of MPO+ NETs at day-1 increase the expected number of days with severe hypoxemia within day-7 by a factor 0.84, holding all other variables constant*.

* *The variable “Lung CT scan lesions” is an ordinal variable treated as follows: <25% (reference), 25–50%, 50–75%, >75%. The incidence rate ratio should be interpreted as the change between two increasing categories*.

### Secondary Outcomes

#### Respiratory SOFA

**Figure 2:**
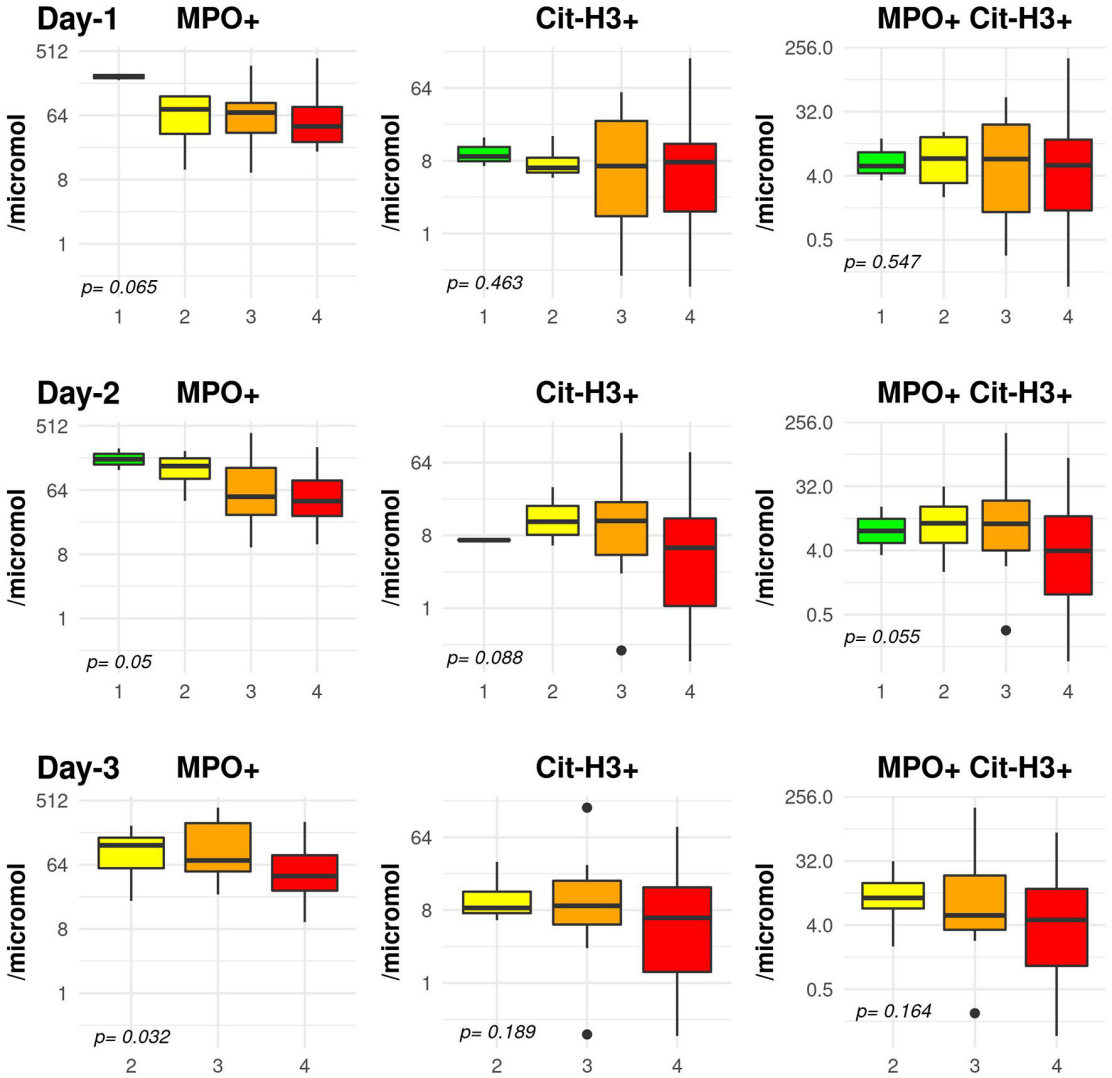
Association between day-1 blood level of NETs and respiratory sequential organ failure assessment (SOFA) score at day-1, day-2, and day-3. The y-axis is shown using a logarithmic scale. The comparison is performed using a Jonckheere test to take into consideration the ordered respiratory SOFA score.

The association between the blood level of NETs at day-1 and respiratory SOFA at day-1, day-2, and day-3 is shown on Figure 2. We observed a significant negative association between MPO+ NETs and SOFA at day-1, 2, and 3 (*p*-value 0.035, 0.044, and 0.015, respectively) while all other levels did not differ over time. Similar observations were made with PaO_2_/FIO_2_ ratio (Supplementary Figure 4).

#### Day-3 Blood Levels of NETs

Overall, MPO+ NETs did not vary between day-1 and day-3 (44.1 [iqr 22.7, 88.6] and 47 [33.9, 66.7], *p* = 0.375) while Cit-H3+ NETs increased and MPO+ Cit-H3+ NETs decreased over time (4.1 [1.5, 10.4] vs. 1 [0, 3.1] *p* < 0.001 and 2.6 [1.1, 7.7] *p* < 0.001, respectively).

#### Day-28 Mortality

**Figure 3:**
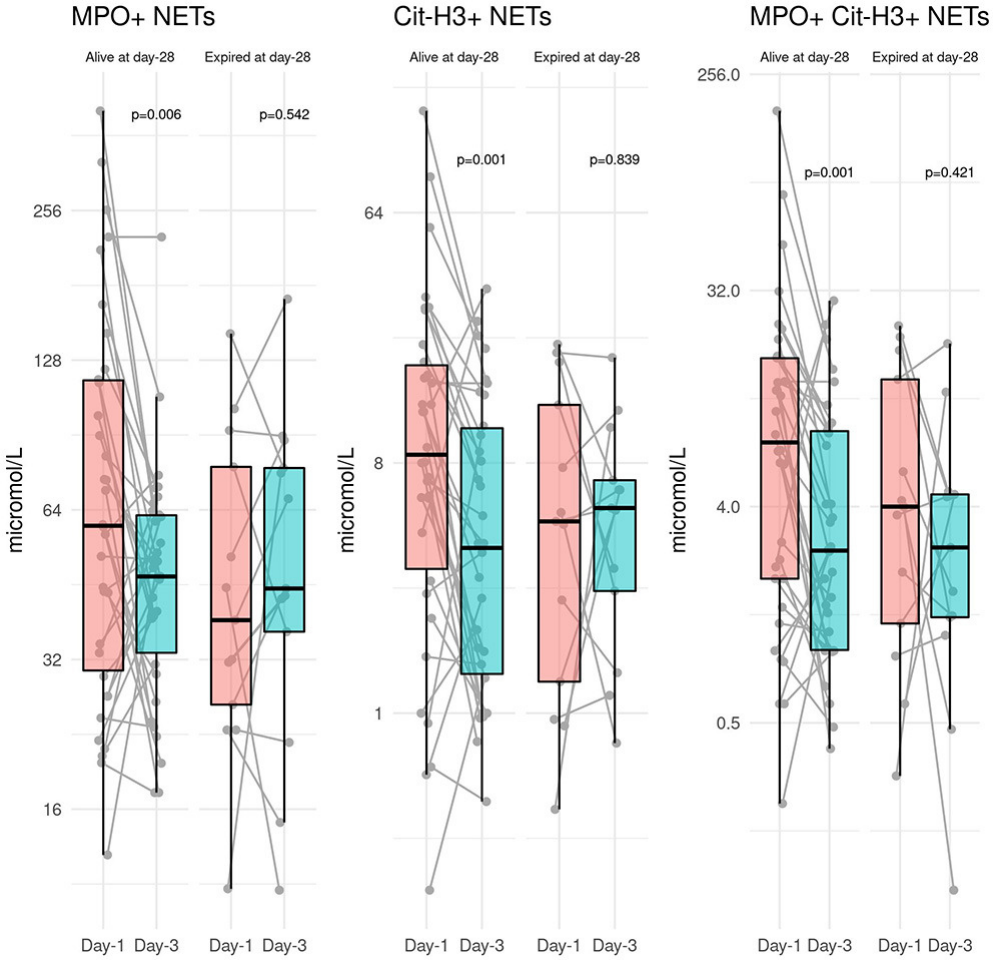
Evolution of blood levels of NETs between day-1 and day-3 according to the vital status at day-28.

Day-28 mortality was 27.6% (*n* = 16). The blood level of NETs significantly decreased between day-1 and day-3 in patients who survived (59.5 [30.5, 116.6] to 47 [33.2, 62.4] *p* = 0.006; 8.6 [3.4, 18.0] to 4 [1.4, 10.7] *p* = 0.001; and 7.4 [4.0, 16.7] to 2.6 [1.0, 8.3] *p* = 0.001 for MPO+, Cit-H3+, and MPO+ Cit-H3+ NETs, respectively). The blood levels of NETs remained stable in patients who died between day-1 and day-3 (38.4 [26.0, 54.8] to 44.5 [36.4, 77.7] *p* = 0.542; 4.9 [1.3, 13.0] to 5.5 [2.8, 6.9] *p* = 0.839; and 4 [1.3, 13.6] to 2.7 [1.4, 4.5] *p* = 0.421 for MPO+, Cit-H3+, and MPO+ Cit-H3+ NETs, respectively) (38.4 [26.0, 54.8] vs. 44.5 [36.4, 77.7]) (Figure 3).

#### Hemostasis and Thrombosis

We observed significant correlations between day-1 blood level of NETs and platelets, prothrombin ratio and fibrinogen level (Supplementary Figures 5–7). Twelve patients (22.2%) received therapeutic-intensity anticoagulation with either low-molecular weight or unfractionated heparin, 11 (20.4%) received double-dose low-molecular weight heparin and 31 (57.4%) received single-dose low-molecular weight heparin. We observed at least one thrombotic event in 14/58 (24.1%) patients included in the analysis. No difference was observed between patients with and without thrombotic events regards to the anticoagulation regimen. Blood levels of NETs at day-1 were significantly lower in patients with compared to those without thrombotic events (39.7 [27.9, 74.4] vs. 47.9 [27.8, 88.6], 0.6 [0.0, 3.6] vs. 2.1 [0.2, 3.3], and 4.1 [0.8, 14.0] vs. 6.8 [2.6, 15.2] *p* < 0.001 for all, for MPO+, HCIT+, and MPO+ Cit-H3+ NETs) (Supplementary Figure 8).

#### Right Ventricular Dysfunction

Blood levels of NETs at day-1 was significantly lower in patients with vs. without a right ventricular dilation (40 [33.5, 105.6] vs. 63 [37.7, 88.6] *p* < 0.001, 0 [0, 0.3] vs. 3 [0.4, 4.9] *p* = 0.622, and 2.7 [0.8, 12.5] vs. 6.1 [2.6, 18.4] *p* < 0.001 for MPO+, Cit-H3+, and MPO+ Cit-H3+ NETs, respectively). Similar results were obtained in patients with vs. without *acute cor pulmonale* (36.3 [33.5, 55.1] vs. 60.3 [36.3, 101.2] *p* < 0.001, 0 [0, 0.1] vs. 2.3 [0.3, 4.6] *p* < 0.001, and 1.3 [0.7, 8.2] vs. 5.8 [1.9, 14.4] *p* < 0.001 for MPO+, Cit-H3+, and MPO+ Cit-H3+ NETs, respectively).

## Discussion

In this prospective observational study including 58 patients admitted to the intensive care unit for acute respiratory failure related to SARS-CoV2 pneumonia, we observed a negative association between the blood level of NETs at day-1 and (1) the severity of the respiratory status, (2) the occurrence of thrombotic events, and (3) the occurrence of right ventricular failure assessed by echocardiography. We also found that a stable level of NETs between day-1 and day-3 discriminated patients who died and those who were still alive at day-28.

### NETs and Severity of Hypoxemia

In the present cohort, whole blood levels of NETs at day-1 strongly correlated with severity of hypoxemia. In multivariable analysis, it was independently associated with the number of days with severe hypoxemia (defined as PaO2/FIO_2_ ratio <100 mmHg). Moreover, the blood level of MPO+ Cit-H3+ NETs in healthy controls was lower than in patients admitted to the ICU for SARS-CoV2 related pneumonia.

Interestingly, these results were not expected. In animal models of influenza pneumonia, the presence of NETs was associated with acute lung injury (12). In human being admitted to the hospital for acute respiratory failure related to influenza, the plasma level of NETs was correlated with the severity of the respiratory status (36). However, in the latter, the plasma cell-free DNA levels did not correlate with PaO_2_/FIO_2_ values but with systemic inflammation (36). Plasma NETs levels were also associated with ARDS severity and mortality in a cohort of 104 ARDS patients (7). Interestingly, Bendib et al. found similar results in a prospective cohort of 35 ARDS patients. Alveolar NETosis in these ARDS patients was inversely associated with hypoxemia and there was no significant association with either day-28 mortality or the number of mechanical ventilation free days (6). Moreover, reduced NETosis under hypoxia has also been evidenced in previous publications (37, 38). In the COVID-19 setting, Middleton et al. reported similar findings in a small sample of COVID-19 patients with a negative relationship between NETosis evaluated using ELISA and severity of hypoxemia (39). Two hypotheses should be discussed to explain this observation. First, this decreased production of NETs could result from a functional defect of polymorphonuclear neutrophils cells, i.e., the number of neutrophils may be normal despite inability to release NETs. Furthermore, such a defect could be explained by the singular inflammatory phenotype of SARS-CoV2 infection in which an impaired type I interferon activity has been shown in a severity-dependent fashion (40). As interferon is a primer of NET production and release (41), the immunological specificities of COVID-19 may at least partly explain the observations we made. Second, we could hypothesize that the blood levels did not accurately reflect what happened in the lung. We were not able to provide sufficient data on alveolar liquid to answer this question. However, in two patients, one with a mild lung injury and the other with a severe one, we observed significantly different amounts of NETs (Supplementary Figure 9) but of course these results are too parcellar to draw robust conclusions. A sequestration in the targeted organ of the virus could be part of the explanation of what we observed.

### NETs and Day-28 Mortality

While the blood levels of NETs at day-1 did not differ between patients dead and alive at day-28, we found that the decrease of blood level of NETs between day-1 and day-3 was strongly correlated with survival to day 28. To the best of our knowledge, this is the first time such a finding is provided. Bendib et al. have already described that blood level of NETs decreased over time in critically ill patients while it remained constant in the bronchoalveolar fluid (8). This was suggestive of a “logical” targeted action of NETs in the lung of patients with pneumonia. However, no description was made in that study of the outcome according to the variations of blood levels of NETs over time. Such a finding could suggest that this lack of decrease in patients with an unfavorable outcome at day-28 reflects either the lack of control of the infection leading to the recruitment of more polymorphonuclear neutrophils and/or the detrimental effect of these NETs in response to the viral aggression. These detrimental effects have already been described in numerous publications. NETs may function as double-edged swords, as they may be a source of immune and pro-inflammatory effectors that may promote tissue damage and autoimmunity (42). In the context of COVID-19, such a mechanism could have amplified the cytokine release syndrome that has been observed in the most severe patients. We are not able to provide further explanation to this finding. However, this seems of interest as this could help to identify patients who could benefit for targeted therapies.

### NETs, Thrombotic Events, and Right Ventricular Failure

In the present study, we also observed a negative association between blood levels of NETs and thrombotic events as well as the occurrence of right ventricular failure. While numerous publications have been reported so far about the high incidence of thrombotic events and right ventricular dysfunction in COVID-19 patients (43, 44), we could have expected a positive association of the blood level of NETs with the occurrence of thrombotic events. Indeed, the level of NETs has been strongly associated with both venous and arterial thrombosis (45, 46). These results could be interpreted in line with a recent publication that provides insights in the role of NETs in thrombosis (47). Noubouossie et al. have shown that NETs had no procoagulant effect *in vitro* while degradation products (such as single histones, purified DNA) activated the intrinsic pathway of coagulation (48). The mechanism explaining this observation remains unclear but this could be related to the neutralization of the negative charge of DNA on the NET surface. Our results add some contribution to these experimental findings, reinforcing the hypothesis that instead of targeting NETs, therapeutic strategies might be focused on components of NETs, leading to a better neutralization of their detrimental effects.

### Limitations

We acknowledge some limitations. First, the present study is observational and what we observed does not imply causality but association. Second, we were not able to provide sufficient data about the level of NETs in the bronchoalveolar fluid. This could be of importance as such results could have been a better reflection of what happened in the lung of patients with mainly a respiratory involvement of COVID-19. However, we believe our results provide significant insights as COVID-19 has been shown a multi-systemic disease with autoimmune and thrombotic symptoms. Third, we did not provide functional assessment of neutrophil functions that could support the hypothesis that the negative association we observed was related to a decreased potential of neutrophils. Last, the evaluation of the occurrence of thrombotic events was made within the first days after ICU admission. This could preclude an accurate evaluation of the relationship between the blood level of NETs at day-1 and such an event that might have occurred a few days later.

## Conclusion

The whole blood level of NETs at day-1 was negatively associated with the number of days with severe hypoxemia in patients admitted to the intensive care unit for SARS-CoV2 related pneumonia. The lack of decrease of the blood level of NETs between day-1 and day-3 discriminated the patients who died within day-28. Whether the NETs could be a therapeutic target in COVID-19 patients should be further investigated.

## Data Availability Statement

The raw data supporting the conclusions of this article will be made available by the authors, without undue reservation.

## Ethics Statement

The studies involving human participants were reviewed and approved by the Research Ethics Board of the Foch Hospital (Suresnes, France, n° 20-04-01) on April 06, 2020. Written informed consent for participation was not required for this study in accordance with the national legislation and the institutional requirements.

## Author Contributions

MG, JZ, VB, and GG: study design. GG: statistical analysis. All authors: acquisition, analysis, or interpretation of the data, and critical revision of the manuscript for important intellectual content.

## Conflict of Interest

The authors declare that the research was conducted in the absence of any commercial or financial relationships that could be construed as a potential conflict of interest.
