# Supplementary material for: Neutrophil Extracellular Traps in SARS-CoV2 Related Pneumonia in ICU Patients: The NETCOV2 Study
Source: Front Med (Lausanne). 2021 Feb 23;8:615984. doi: 10.3389/fmed.2021.615984 (PMC7940514; doi:10.3389/fmed.2021.615984)
Supplement: Supplementary file 1 [file Table_1.DOCX]

**NETosis in SARS-CoV2 related pneumonia in ICU patients: the NETCOV2 study**

Mathieu Godement, Jaja Zhu, Charles Cerf, Antoine Vieillard-Baron, Agathe Maillon, Benjamin Zuber, Valérie Bardet, Guillaume Geri

**Supplementary material**

eFigure 1 Blood levels of NETs at day-1 and of blood leucocytes at day-1 (top) and day-2 (bottom). Each point shows a patient. The colour shows the respiratory SOFA score (purple, blue, green and yellow for 1,2,3 and 4, respectively). Pearson’s correlation statistics are shown on each plot.


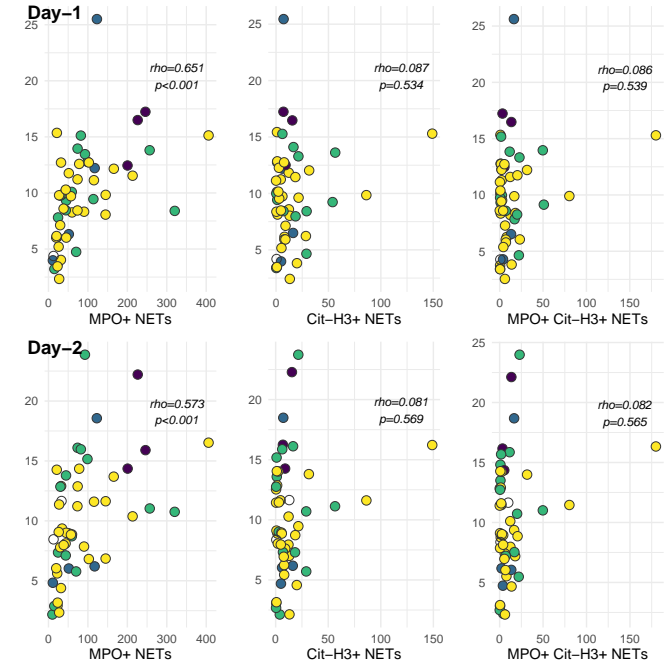


eFigure 2 Blood levels of NETs at day-1 and of blood polymorphonuclear neutrophils at day-1(top) and day-2 (bottom). Each point shows a patient. The colour shows the respiratory SOFA score (purple, blue, green and yellow for 1,2,3 and 4, respectively). Pearson’s correlation statistics are shown on each plot.


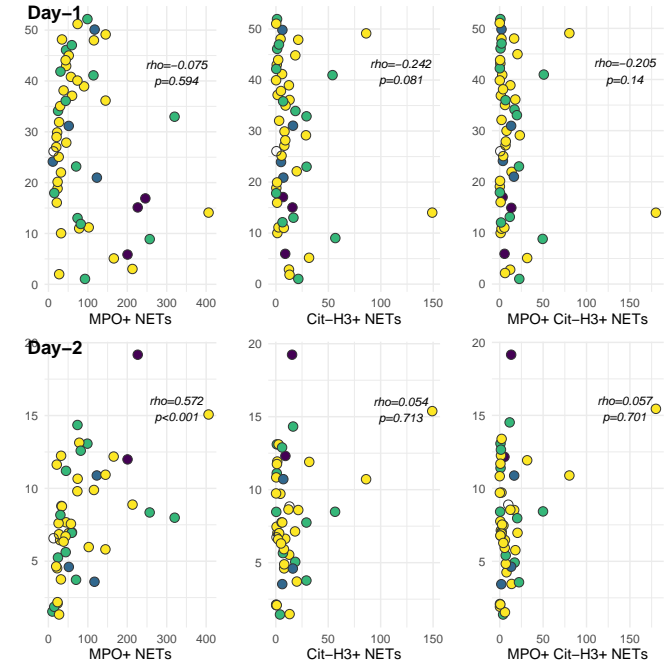


eFigure 3 Comparison of the blood level of NETs from healthy controls and SARS-CoV2 patients (at day-1). Comparison has been made using a Mann-Whitney test.


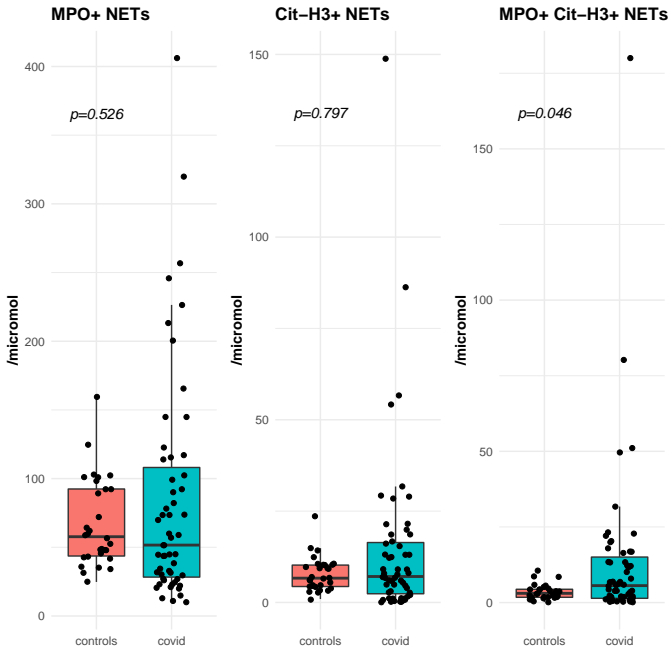


eFigure 4 Association between PaO2/FiO2 ratio and blood levels of NETs at day-1, day-2 and day-3. The colour shows the respiratory SOFA score (purple, blue, green and yellow for 1,2,3 and 4, respectively). Pearson’s correlation statistics are shown on each plot.


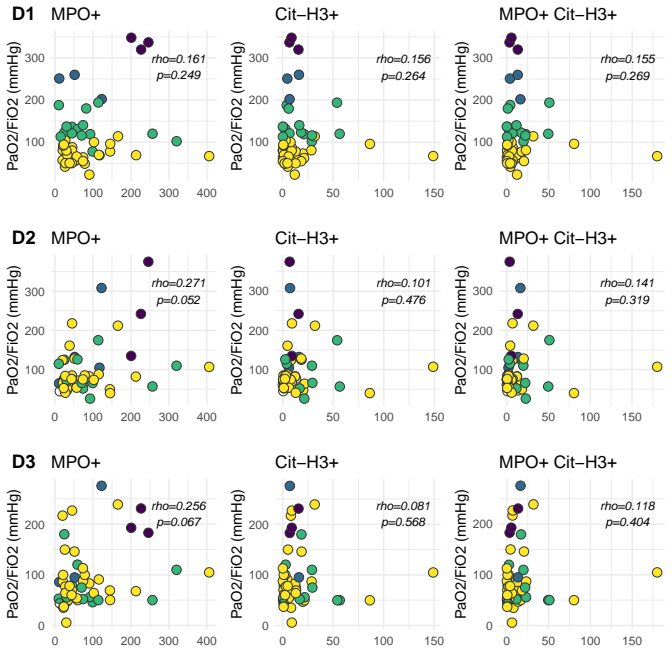


eFigure 5 Correlation between haemostasis and day-1 blood level of MPO+ NETs


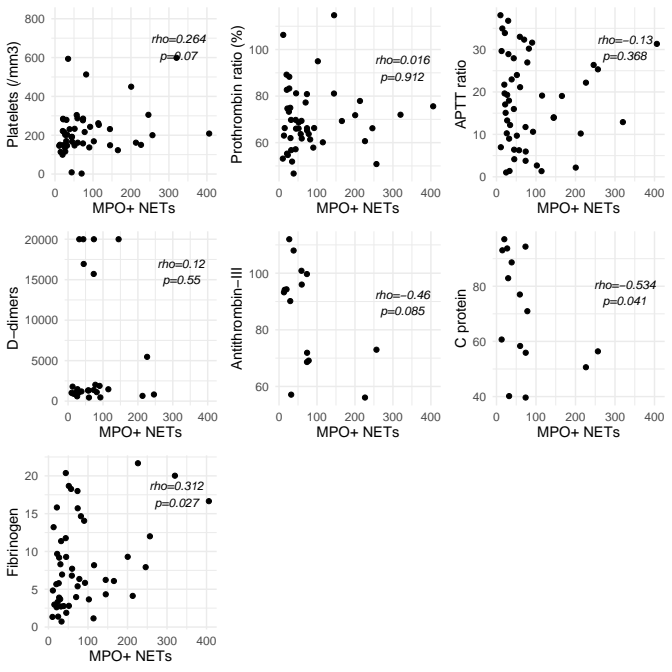


eFigure 6 Correlation between haemostasis and day-1 blood level of Cit-H3+ NETs


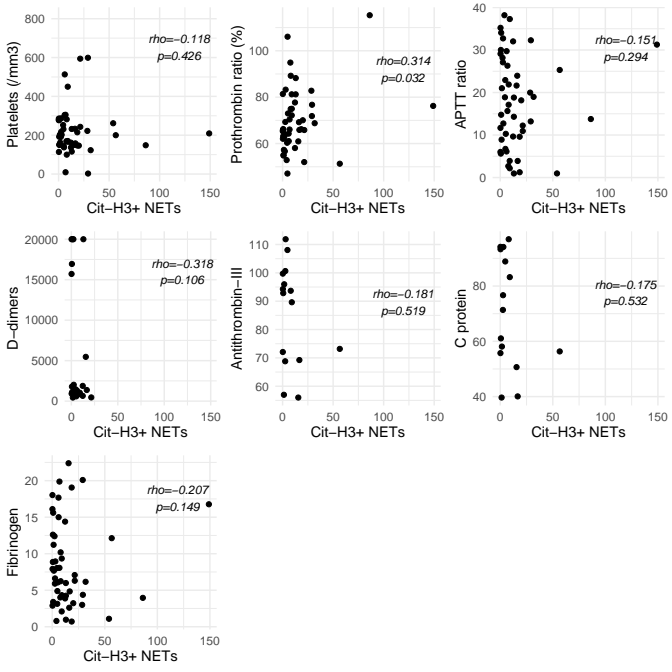


eFigure 7 Correlation between haemostasis and day-1 blood level of MPO+Cit-H3+ NETs

*
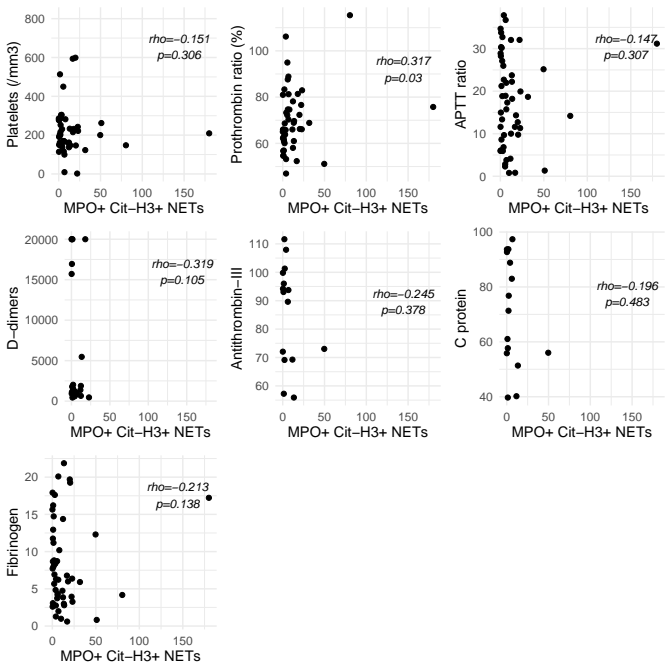
*

eFigure 8 Blood levels of NETs at day-1 according to thrombotic events

eFigure 9 Typical staining BAL staining in two patients.

Panel A on the top shows BAL staining from a hypoxemic patient with a mild lung injury and panel B shows BAL from a patient with a severe lung injury. Despite analysis of relatively similar numbers of nucleated cells (Leukocytes gate), NET events were around ten times more frequent in patient B, suggesting that NETs were trapped in the lung as discussed in the paper.


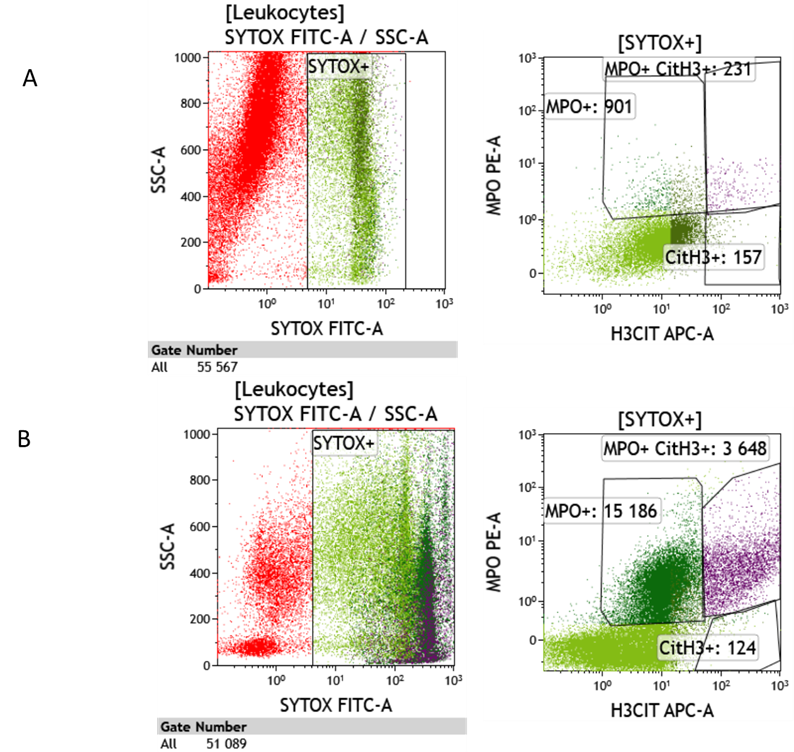


eTable 1 Blood levels of NETs at day-1 across tertiles of number of days with severe hypoxemia within day-7

|  | Number of days with severe hypoxemia within day-7 | | | P value |
| --- | --- | --- | --- | --- |
|  | [0, 3[ days | [3, 6[ days | [6,7] days |  |
| MPO+ NETs | 63.0 [14.5, 133.9] | 40.7 [25.0, 76.0] | 43.9 [22.1, 66.5] | 0.674 |
| Cit-H3+ NETs | 2.0 [0.3, 3.4] | 0.7 [0.0, 3.0] | 0.9 [0.0, 4.8] | 0.693 |
| MPO+ Cit-H3+ NETs | 4.7 [2.4, 13.3] | 4.9 [1.3, 13.2] | 5.8 [1.0, 15.2] | 0.956 |

eTable 2 Number of days with severe hypoxemia within day-7 according to quartiles of blood levels of NETs at day-1

|  | Quartile 1 | Quartile 2 | Quartile 3 | Quartile 4 | P value |
| --- | --- | --- | --- | --- | --- |
| MPO+ | 4 [2, 6] | 4.5 [4, 6] | 4 [4, 6] | 2.5 [0, 5.8] | 0.12 |
| Cit-H3 + | 4 [2.5, 6] | 5 [4, 6.3] | 3 [2, 5] | 4 [0.5, 6] | 0.63 |
| MPO+ Cit-H3+ | 6 [4, 6] | 2.5 [0, 4] | 4 [4, 6] | 3.5 [2, 6] | 0.59 |

Comparison of ordered categories has been performed using a Jockheere test
